# Sequestration and Transport of Lignin Monomeric Precursors

**DOI:** 10.3390/molecules16010710

**Published:** 2011-01-18

**Authors:** Chang-Jun Liu, Yu-Chen Miao, Ke-Wei Zhang

**Affiliations:** Biology Department, Brookhaven National Laboratory, Upton, NY 11973, USA

**Keywords:** lignin, monolignols, monolignol glucosides, ABC transporters, vacuolar sequestration

## Abstract

Lignin is the second most abundant terrestrial biopolymer after cellulose. It is essential for the viability of vascular plants. Lignin precursors, the monolignols, are synthesized within the cytosol of the cell. Thereafter, these monomeric precursors are exported into the cell wall, where they are polymerized and integrated into the wall matrix. Accordingly, transport of monolignols across cell membranes is a critical step affecting deposition of lignin in the secondarily thickened cell wall. While the biosynthesis of monolignols is relatively well understood, our knowledge of sequestration and transport of these monomers is sketchy. In this article, we review different hypotheses on monolignol transport and summarize the recent progresses toward the understanding of the molecular mechanisms underlying monolignol sequestration and transport across membranes. Deciphering molecular mechanisms for lignin precursor transport will support a better biotechnological solution to manipulate plant lignification for more efficient agricultural and industrial applications of cell wall biomass.

## 1. Introduction 

Lignin is the second most abundant terrestrial biopolymer after cellulose, accounting for approximately 30% of organic carbon in the biosphere [1]. It is a crucial component in preserving the structural integrity of plant cell wall, therefore, affording stiffness and strength of the stem of vascular plants; enabling transport of water and solutes through tubes in the vascular system; and physically protecting plants against pathogen infection and other environmental stresses [1,2]. However, its presence in cell wall greatly contributes to the wall’s recalcitrance to hydrolysis, thus detrimental to the applications of cellulosic fibers for pulping and biofuel production [3,4,5]. Plant lignification is considered to occur in three stages: biosynthesis of monolignols in the cell, transport of monolignols to the cell wall, and dehydrogenative polymerization of monolignols within the cell wall. Although extensive studies have centered on lignin biosynthesis for many decades, several fundamental aspects on plant lignification remain elusive. One of the most intriguing yet least understood aspects concerns the intracellular sequestration of lignin precursors and their transport across the plasma membrane. In this review, we outline the hypothesized mechanisms for monolignol sequestration and transport, and describe the recent progresses in genetic, transcriptomic, proteomic, modern autoradiographic, and biochemical studies toward understanding the underlying molecular mechanisms for monolignol sequestration and transport across membranes. An improved knowledge of transport of monolignols will facilitate the efficient manipulation of plant ligninfication for utilization of lignocellulosic biomass. 

## 2. Results and Discussion 

### 2.1. Monolignol biosynthesis and subcellular localization of the related enzymes

Lignins are complex racemic aromatic heteropolymers derived mainly from three hydroxycinnamyl alcohols, *i.e.*, *p*-coumaryl, coniferyl and sinapyl alcohols [2]. Biosynthesis of monolignols diverges from the shikimate pathway. Starting from phenylalanine, monolignol biosynthesis is sequentially catalyzed by about 10 enzymes [4], namely phenylalanine ammonia lyase (PAL), cinnamic acid 4-hydroxylase (C4H), 4-hydroxycinnamoyl CoA ligase (4CL), hydroxycinnamoyl CoA:shikimate hydroxycinnamoyl transferase (HCT), *p*-coumaroylshikimate 3΄-hydroxylase (C3΄H), caffeoyl CoA *O*-methyltransferase (CCoAOMT), hydroxycinnamoyl CoA reductase (CCR), ferulic acid 5-hydroxylase (F5H), caffeic acid/5-hydroxyferulic acid *O*-methyltransferase (COMT), and (hydroxy)cinnamyl alcohol dehydrogenase (CAD) [1]. The activities of these enzymes lead to deamination, hydroxylation, transacylation, methylation, and reduction of phenylalanine, and transform it to the lignin monomeric precursors, *p*-coumaryl, coniferyl, and sinapyl alcohols (*i.e.*, monolignols) [1,4]. Among these enzymes, three cytochrome P450 proteins, *i.e.*, cinnamic acid 4-hydroxylase (C4H), *p*-coumaroylshikimate 3΄-hydroxylase (C3΄H), and ferulic acid 5-hydroxylase (F5H) are membrane-bound proteins and associate with the outer surface of the endoplasmic reticulum by virtue of their N-terminal membrane anchor [4]. Nevertheless, most other enzymes, such as PAL, 4CL, COMT, CCoAOMT and CAD in diverse species are soluble and localize within cytosol that were revealed by biochemical and immuno-cytochemical studies, although a few studies suggested that PAL and CAD may have different types of isoforms, and some of them may associate to the ER-Golgi derived vesicles [6,7]. For example, one tobacco PAL isoform, when fused with GFP protein, was found localized to microsomal fractions, where it co-localized with C4H, forming a potential metabolic channeling at the entry point of phenylpropanoid-lignin biosynthetic pathway [8]. 

The cytosolic localization of those biosynthetic enzymes suggests that monolignols are synthesized in the cytoplasm. After that, they need to be transported to the cell wall where they are oxidatively polymerized, and integrated into the secondary cell-wall matrix.

### 2.2. Monolignol glucosylation and its potential roles in sequestration and transport of monolignols 

In gymnosperms and some angiosperm species, monolignols are often glycosylated to form 4-*O*-β-d-glucosides, namely, coniferin and syringin [9]. Assessing monolignol glucoside content in several dicot trees and gymnosperm species from different genera, Terazawa *et al*. found that the monolignol glucosides (coniferin) were present primarily in gymnosperm species [10]. Leinhos and Savidge also showed that coniferin present at high level within protoplasts made from the developing xylems of Jack pine (*Pinus banksiana*) and Eastern white pine (*P. strobes*) [9]. The coniferin content of the protoplasts can account for most, if not all, of the coniferin present in the tissue. This leads to the assumption that the high level-accumulated monolignol glucosides most likely are stored in vacuoles of cambial cells [11,12,13,14]. 

**Figure 1:**
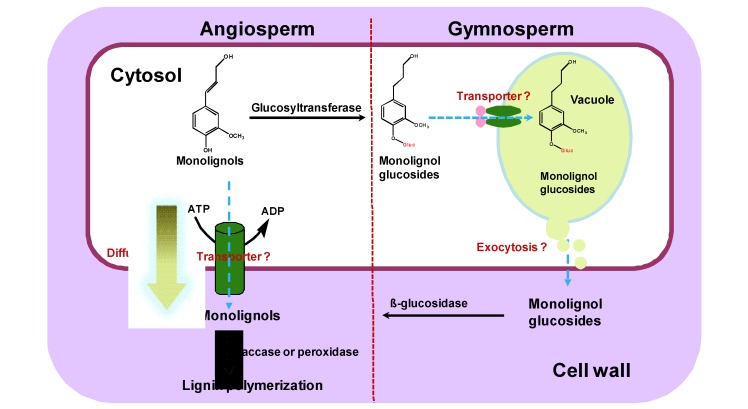
Alternative models or pathways of monolignol transport in angiosperms and gymnosperms.

The possible presence of monolignol glucosides in vacuoles of differentiating conifer xylem cells led to the suggestion that at least in gymnosperms and some evolutionarily less-derived angiosperm species such as *Magnoliaceae* and *Oleaceae* families, monolignol glucosides may be the storage or transport forms of the monolignols; and the lignin biosynthesis and mobilization of the synthetic precursors in these species may be regulated by monolignol glucosides. The glucoconjugates of monolignols are possibly sequestrated first from cytosol to vacuole then be transported from vacuole to cell wall through an unknown mechanism (Figure 1) [14,15,16,17]. 

Monolignol glycosylation is catalyzed by soluble UDP-glucose: coniferyl alcohol or sinapyl alcohol glucosyltransferase [18]. In a survey conducted by Ibrahim [19], coniferyl alcohol glucosyltransferase activity was detected in crude homogenates of all gymnosperms tested; among angiosperms, woody species exhibited higher enzyme activities than herbaceous species [19]. On the other hand, the specific β-glucosidases are proposed to release monolignols from the glucoconjugates in cell wall of differentiated xylem tissues. The corresponding enzymes have been characterized from both gymnosperm and angiosperm sources, such as spruce seedlings [20], chick pea cell suspension cultures and seedlings [21], soybean cell cultures, hypocotyls and roots [22], the differentiating xylem of Jack pine [23] and lodgepole pine [14]. Moreover, the apoplastic location of β-glucosidases was demonstrated by immunohistochemical techniques [24,25]. Consequently, the cDNAs encoding coniferin β-glucosidase have been isolated from both lodgepole pine (gymnosperm) and Arabidopsis (angiosperm). Their expression was proved highly specific in the site of lignifications [15,17]. Based on those evidences, a UDPG:coniferyl alcohol glucosyltransferase/coniferin β-glucosidase (CG) system was proposed to regulate the storage and mobilization of monolignols for lignin biosynthesis and plant lignification [14,15,16,17].

A small cluster of UDPG-glycosyltransferase genes were functionally characterized from *Arabidopsis*. The encoded enzymes were able to 4-*O*-glucosylate monolignols and other phenolics *in vitro* [26]. Down- or up-regulation of those identified glucosyltransferases resulted in the corresponding reduction or accumulation of the soluble monolignol glucosides in the transgenic roots or leaves of *Arabidopsis* [27]. However, no significant changes of lignin deposition was resulted from the disturbance of those glucosyltransferase genes’ expression [3]. These data suggest that monolignol glucosylation might not play a role in exporting monolignols, at least in angiosperm species.

Moreover, a recent experiment feeding [^3^H]-Phe to the dissected xylem of lodgepole pine showed that the radiolabel was incorporated directly into monolignols and to the lignin constituents accumulated in the cell wall. Neither substantial amount of radiolabel was associated with the glucoside of monolignol (coniferin), nor with the interior of the central vacuole, where coniferin is expectedly stored [28]. These data further argue against the role of monolignol glucosylation in the export of monolignols across the plasma membrane and imply that monolignol aglycone may be the chemical form for the transport.

Monolignols are relatively toxic, and unstable. Glucosylation of small molecule compounds is known to reduce their lipophilicity, thus preventing any further possibility of free diffusion across the lipid bilayer [29]. Therefore, the role of monolignol glucosylation may be to convert the highly active, unstable lignin precursors into the “storage form”, as precursor reservoirs shielded them in a particular compartment. 

### 2.3. Mechanisms for the transport of monolignols across membranes

The exact mechanism of monolignol transport from cytosol to cell wall remains unclear, although there are several proposed models or pathways. Early studies involving chemical analysis, radiotracers and microscopic examination suggested different mechanisms explaining the transport of monolignols (Figure 1). 

#### 2.3.1. Exocytosic transport via the Endoplasmic Reticulum- Golgi derived vesicles 

The non-cellulosic polysaccharides of plant cell wall, such as pectin and hemicelluloses, are well defined to be synthesized within the Golgi bodies and exported to the cell wall through an exocytosis mechanism [30]. Early autoradiographic, immunocytochemical, and ultrastructural studies suggested that transport of lignin precursors was also potentially via a similar ER-Golgi route as does the secretion of the wall matrix polysaccharides. A vesicle trafficking between cytosol and plasmalemma in differentiating tracheids of wheat xylem tissues was observed in the autoradiographic and ultrastructural studies [31]. Employing [^3^H]-Phe, -tyrosine and -cinnamic acid to label the developing xylem, investigators found the radiolabels associated with the rough endoplasmic reticulum, the Golgi apparatus, and with some vesicles fused with the plasma membrane or aggregated in the cytoplasm near the bands of wall microtubules [6,31,32]. These findings suggest that the ER-Golgi apparatus is involved in the synthesis and transport of monolignols to the cell wall. Immunocytochemical localization of PAL and CAD in tracheary elements of *Zinnia elegans* also suggested that the immunolabels of PAL and CAD seemed not only to disperse in the cytoplasmic matrix, but also localize to the Golgi-derived vesicles and the developing secondary thickenings [7]. These early observations led to the assumption that monolignols as the content of Golgi-derived vesicles could be exported to the cell wall by fusion of vesicular-membrane with the plasmalemma. However, in those early autoradiographic studies, most of the radioactive intermediates of monolignols, might be extracted during the exchange of organic solvents for embedding the specimens in resin. Moreover, the radioactive lignin precursors such as Phe are assimilated efficiently to both lignin and proteins. These could interferer the interpretation of autoradiography. In a recent study, Kaneda *et al.* [28] adopted cryofixation and freezing substitution to prepare the labeled lodgepole pine xylem cells. These new cytological techniques substantially minimized the damage on the sectioned cells, thus preventing pseudo-autographic images. Using the [^3^H]-Phe radiotracer to feed the dissected developing xylem tissue of the lodgepole pine, together with the selective inhibition of phenylpropanoid and protein biosynthesis, respectively, they discovered that the Phe-radiolabel in ER-Golgi was primarily incorporated into proteins, not monolignols. Further, they found that the abundant Golgi and Golgi-vesicle clusters that were observed in the developing xylem cells did not load with phenylpropanoids. Therefore, these up-to-date autoradiographic results suggest that ER-Golgi-vesicle mediated exocytosis is unlikely to play a major role in monolignol transport. 

#### 2.3.2. Passive diffusion

Genetic engineering and chemical compositional analyses revealed that lignin biosynthesis displays considerable plasticity. Besides three classical monolignols (*p*-coumaryl, coniferyl, and sinapyl alcohols), several other phenolic components are also incorporated into lignin polymer. These include the products from the incomplete monolignol biosynthesis, such as 5-hydroxyconiferyl alcohol [1], hydroxycinnamaldehydes [33] and hydroxycinnamic acids [34,35], as well as the enzymatic derivatives of monolignols, such as sinapyl *p*-hydroxybenzoate, coniferyl and sinapyl *p*-coumarate [36], and coniferyl and sinapyl acetate [37,38]. Such accommodation of alternative monomers in ligninifcation infers a potential non-specific passive diffusion of lignin precursors across the plasma membrane [3]. This passive diffusion notion was further evident from the *in vitro* partitioning experiments by using immobilized liposomes or lipid bilayer discs as the model membranes. When lignin precursor analogs were incubated with liposomes or lipid bilayer discs, they were observed to be easily partitioned into the artificial membrane phase [39,40]. However, there are a variety of phenolics synthesized in the cytosol of a living cell, and all have a similar hydrophobic nucleus (phenyl ring), it is not clear how monolignols and/or their particular derivatives are selected and diffuse across the plasma membranes. 

#### 2.3.3. Transporter-mediated monolignol sequestration and export

Many studies show that lignin monomers are differentially deposited in discrete regions of various types of lignifying cell wall. For example, lignin in the vessel cell walls of birch wood is derived mainly from coniferyl alcohol, whereas the fiber wall incorporates both sinapyl and coniferyl alcohols [41]. Similarly, in *Arabidopsis* stems, the lignin of the vascular bundle rich in vessels primarily contains G units, while the interfascicular fibers are enriched in S units [42]. In spruce wood, the lignin of the middle lamella embeds more *p*-coumaryl alcohol units than does the secondary wall lignin that was mainly derived from coniferyl alcohol [43]. When feeding the labeled monolignols into developing xylem, the radiolabeled *p*-coumaryl alcohol was preferentially deposited in the middle lamella/cell corners, whereas coniferyl alcohol was mainly located in the secondary wall [44]. Moreover, during development of secondary cell wall to form S1-, S2-, and S3- layers, the three kinds of monolignols exhibited sequential deposition, in the order of *p*-coumaryl alcohol (for H unit), coniferyl alcohol (G unit) and sinapyl alcohol (S unit) [44,45,46]. These data suggest that lignin monomer deposition into cell wall is a highly organized, regulated process, and that some active transport mechanisms may selectively permit the deposition of the particular monolignols.

Recent functional genomic, global transcriptomic and proteomic studies in both gymnosperms and angiosperms frequently uncovered some putative membrane transporters potentially involved in xylem differentiation, and secondary cell-wall formation. They are highly expressed in the lignified wood tissues. For example, a number of ESTs encoding for ATP-binding cassette (ABC) transporters were disclosed from several studies on gene expression during wood transformation [47,48,49,50]. When analyzing the data of the first generation of loblolly pine microarray, the distinct ABC transporter-like genes were found abundant in the mature wood and compression wood, wherein different types of lignins accumulated [50,51]. Similarly, a set of ABC transporters were identified in a microarray study of gene expression during lignification of Arabidopsis stem. Those ABC transporters were coordinately expressed with the known lignin biosynthetic enzymes [52,53]. In a recent proteomics study on plasma membrane from poplar leaf, xylem and cambium/phloem tissues, a set of ABC transporters showed a rather “specific” tissue distribution [54]. One member in the subfamily B and three in G were identified in cambium/phloem only; while a member of subfamily B was specific to xylem, and its *Arabidopsis* homolog, ABCB15 (MDR13), was correlated well with lignin biosynthetic genes in a transcritpomic studies [52]. These data imply that membrane transporters, particularly, ABC transporters, might have a significant role in cell wall lignification, presumably in monolignol transport to the cell wall.

### 2.4. Membrane transporters and their implication in phenolic transport 

Sequestering small molecule metabolites within particular intracellular compartments or transferring them between different cell types require specific multidrug transporters. The multidrug transporters form a large class of membrane proteins present in the cells of most organisms. These transporters fall into five families: The ATP-binding cassette superfamily (ABC), the major facilitator superfamily, the small multidrug resistance family, the resistance-nodulation-cell division family, and the multidrug and toxic compound extrusion (MATE) family [55]. 

The ABC transporters constitute a large, diverse, and ubiquitous superfamily. The majority of ABC genes encode membrane-bound proteins that participate in transporting a wide range of molecules across membranes [56,57,58]. The ABC proteins can be broadly categorized as importers or exporters, depending on the direction of movement relative to the cytoplasm [59]. According to a recent inventory and new nomenclature for ABC proteins, they can be divided into nine subfamilies, namely ABCA–I, with all subfamilies except H found in plants [58]. ABC proteins are defined by their possession of an ATP-binding cassette, also known as the nucleotide-binding domain (NBD)[58,60]. In addition to NBDs, ABC transporters also encompass transmembrane domains (TMDs), each composed of several hydrophobic α-helices. The core units comprising a functional ABC transporter consist of four domains: Two NBDs, and two TMDs. The former cooperatively bind and hydrolyze ATP, providing the driving force for transport, and the latter are involved in substrate recognition and translocation across the lipid bilayer. Although most prokaryotic ABC proteins are encoded as the separate TMD and NBD subunits or as “one-half-size” (one TMD and one NBD) transporters, in the majority of eukaryotic ABC transporters, all four domains (two TMDs and two NBDs) are contiguous on a single polypeptide, constituting a full length transporter in the “forward” (TMD1-NBD1-TMD2-NBD2) orientation, or the “reverse” (NBD1-TMD1-NBD2-TMD2) orientation. In a particular subfamily, the ABCC (MRP), the arrangement is in the forward orientation, but some proteins, e.g., MRP1 have extra five α helices (sometimes designated as TMD0) at the N terminus [59,61].

The *Arabidopsis* and rice (*Oryza sativa*) genomes each contains more than 120 putative ABC transporters, but only a few have been characterized [62]. These proteins show diverse transport activities to a variety of small molecule metabolites, such as glutathione-conjugates [63,64], chlorophyll catabolites [63,64], peptides [65], lipids [66], auxins [67,68,69], abscisic acid [70,71], inorganic ions [72], malate [73], wax and cutin precursors [74,75], defensive secondary metabolites such as antifungal terpenoids [76], tropane alkaloids (hyoscyamine and scopolamine) [77], benzylisoquinoline alkaloid (berberine) [78,79], and xenobiotics [80]. 

Among these identified ABC transporters, a few are revealed responsible for phenolic/polyphenolic transport [81]. A membrane-potential dependent ABC-like transporter was demonstrated to mediate the vacuolar uptake of flavone glucuronides in rye [82,83]. Genetic suppression of the maize tonoplast-localized GS-X pump, the ZmMRP3, resulted in the reduced anthocyanin accumulation [84], although the transport activity of ZmMRP3 to anthocyanin has yet to be shown. In addition, plasma membrane ABC transporter-mediated secretion of the isoflavone genistein in soybean roots has been reported [85], but the gene encoding the protein is yet to be identified. 

Besides involvement of the energized primary transporter, *i.e.*, ABC transporters, transporting or sequestering plant small molecule metabolites is also mediated by active secondary transport systems [86]. The MATE family is the most recently classified multidrug resistance-conferring, secondary transporter family [55]. These proteins use an electrochemical gradient (usually H^+^/ Na^+^) as the driving force to transport other substrates across membranes. Their function and activity largely depend on various types of H^+^-ATPases [81]. The MATE family is divided into three groups based on sequence relatedness. Prokaryotes have members of the two more related groups, whereas the third group is composed exclusively of proteins from eukaryotes. Members of this third group of MATE proteins are encoded by about 56 genes in the *Arabidopsis* genome [87]. These genes often are organized in tandem repeats in the genome. 

Biochemical analyses of plant MATE family members suggest these proteins function as efflux pumps. Several MATE transporters have been characterized. These include *Arabidopsis* ALF5 that exports toxic cations such as tetramethylammonium thus protecting roots from exogenous inhibitory compounds [88], AtDTX1 that confers the efflux of toxic compounds norfloxacin, berberine and cadmium tolerance when expressed in *Escherichia coli* [89], and the *Arabidopsis* MATE transporter TT12 that acts as a cyanidin-3-*O*-glucoside/H^+^-antiporter mediates vacuolar sequestration of anthocyanins [90,91]. When expressed in yeast, TT12 and its ortholog MATE1 from *M. truncatula* preferentially transport epicatechin 3΄-*O*-glucoside, the proposed precursor for the synthesis of proanthocyanidins [92]. Therefore, TT12 and its homologs are also responsible for the accumulation of proanthocyanidins in *Arabidopsis* and legume seed coats. Recently, two MATE-type transporters from grapevine are characterized as vacuolar transporters of acylated anthocyanins [93]. In addition, a tobacco MATE homolog is demonstrated responsible for vacuolar sequestration of nicotine alkaloid in the aerial parts of tobacco [94,95]. 

Although both the ABC and MATE membrane-transporters are reportedly involved in the vacuolar sequestration or transport of a range of phenolic glycosides and glutathione conjugates, the transport activity of two types of membrane transporters to small molecule compounds in plant cells displays disparate characteristics [87,96]. Collectively: 1) different glucoconjugates are moved by different transporters using distinct mechanisms. For instance, a flavonoid glucoside, isovitexin, a native C-glucoside in barley, was conveyed into the isolated vacuoles of barley via electrochemical gradient-dependent secondary transport. In contrast, the herbicide glucoside of hydroxyprimisulfuron was taken up by the directly energized primary transport mechanism [97]. 2) Identical compounds under distinct circumstances (e.g., intrinsic or heterologous) in cells may be transported by different mechanisms. The uptake of the main barley flavonoid saponarin, an apigenin glucoside, into barley vacuoles occurred via a proton pump, whereas the transport of the same compound into Arabidopsis vacuoles, a heterologous plant that does not generate this metabolite, displayed typical characteristics of an ABC transporter-mediated process [83]. Moreover, the conveyance of structurally similar endogenous metabolites also necessitates distinct transporters. Transport of anthocyanin into vacuoles in maize involved ABC transporter MRPs [84]. However, sequestration of proanthocyanidins, a group of polyphenolics structurally related to anthocyanidins, requires H^+^-gradient dependent MATE transport [92]. The *Arabidopsis* mutant *transparent testa12* (*tt12*), lacking the gene encoding a MATE family secondary transporter-like protein exhibited strongly reduced proanthocyanidine deposition in the vacuoles of endothelial cells [90,91]. 3) Transport of phenolics is affected by the presence of the hydrophilic ligand (e.g., glucose or glutathione). The uptake of phenolic glucosides of *p*-hydroxy-cinnamic acid and *p*-hydroxybenzoic acid by vacuolar vesicles purified from red beet (*Beta vulgaris*) seemingly was via an H^+^- gradient-dependent transport mechanism [98]. In contrast, when cinnamic acid was conjugated with glutathione, it was transported into the vesicles via a GS-X pump, defined as ABCC protein [99]. These data imply that the sugar moiety may be a preferred ‘tag’ recognized by the secondary transporters, whereas the glutathione moiety is preferred by MRP (ABCC) proteins [100,101] and 4) a given phytochemical can be transported by more than one membrane transporters, on the other hand, a transporter can involve in the secretion of more than one secondary metabolite [102].

### 2.5. ABC-like transporters are involved in the transport of monolignols and their glucosides across membranes

**Figure 2:**
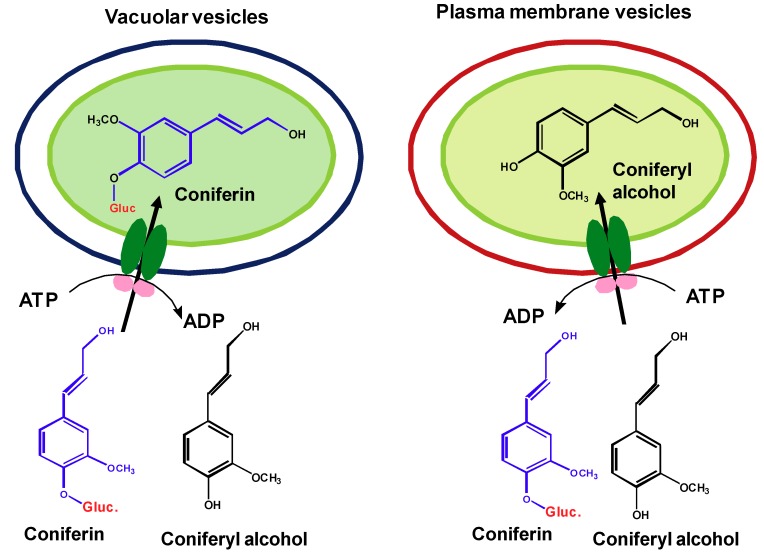
ATP-dependent, selective uptake of monolignols or their glucosides by vacuolar vesicles and the inside-out plasma membrane vesicles.

Although several pieces of information suggest the potential involvement of membrane transporters in the sequestration and transport of lignin precursor across membranes, the direct biochemical evidence has been missing. To clarify whether the transport of lignin precursor is the membrane transporter-mediated active process, exploration was recently conducted in authors’ group by the *in vitro* uptake assay that incubates the isolated plasma and vacuolar membrane vesicles from both *Arabidopsis* young rosette leaves and the roots of poplar with monolignols and/or their glucosides [103]. Our studies demonstrate that the transport of lignin precursors across both plasma and vacuolar membranes is largely dependent on the presence of ATP. In the absence of ATP, the transport activity of either plasma or vacuolar membrane vesicles to monolignols or their glucosides is severely impaired, only 20~30% of that detected in the presence of ATP. Among different nucleotide triphosphates, ATP mostly promotes the transport activity. The specific ABC-type transporter inhibitors largely reduce the transport activity of plasma or vacuolar membrane vesicles to lignin precursors. Furthermore, in the presence of ATP, the vacuolar membrane vesicles prepared from *Arabidopsis* rosette leaves display considerable activity in sequestering the glucoconjugates coniferin and syringin, but a very limited activity to monolignol aglycones. In contrast, the plasma membrane vesicles are inactive to the glucoconjugated monolignols and prefer for the aglycones (Figure 2). These data suggest that glucosylation of monolignols is a prerequisite for their vacuolar sequestration while the aglycones are required for the direct transport into cell walls of *Arabidopsis.* The current studies complement previous genetic observation, wherein down- or up-regulating the expression of *Arabidopsis* UDPG: monolignol glucosyltransferases entailed the corresponding reduction or accumulation of the soluble monolignol glucosides in transgenic roots or leaves [27], but a change in lignin content or composition was not observed. 

The different chemical forms of monolignols required in the ATP-dependent sequestration and transport also implicates the distinct classes of ABC transporters involved in diverting and partitioning the polarized “storage form” glucosides of monolignols into vacuoles, and the hydrophobic aglycones across plasmalemma. In the assay, we also observed that compared to vacuolar vesicles, plasma membranes displayed notable promiscuity in conveying different phenolics in the presence or absence of ATP molecules. The less selectivity of plasma membranes might explain the observed plasticity of lignin biosynthesis. The promiscuous active transport and/or the low level of intrinsic plasmalemma diffusion may lead to the deposition of non-classic lignin precursors into the cell wall.

Although ABC transporter inhibitors severely disrupt the uptake of plasma and vacuolar membrane vesicles to monolignols or their glucosylated derivatives in the uptake assays, ionophoric agents, such as gramicidin D, nigericin and NH_4_Cl that disturb the activities of MATE family transporters, showed less inhibitory effect on the uptake of lignin precursors. After ionophore treatments, both plasma and vacuolar membrane vesicles remained 60~80% of the transport activity in the presence of ATP. These data imply that the secondary energized MATE transporters may not play a vital role as the ABC-type transporters do in mediating lignin precursor transport. This observation is also consistent with those transcriptomics and proteomics studies on cell wall formation. Analyzing global gene expression and examining the integral plasma membrane proteins in differentiated xylem tissues did not reveal particular MATE transporters correlated with lignin biosynthesis [52,54]. 

## 3. Conclusions and Perspectives

Transport of lignin monomers across cell membranes is the critical, albeit least investigated, step in plant lignification. The up- to-date information indicates that: (1) glycoconjugates of monolignols may not be the required transport form across the plasma membranes, although lignin monomeric precursors in all gymnosperms and a few angiosperms are often glucosylated. Like many other stored phenolics, glucosylation of monolignols may simply be to facilitate the vacuolar sequestration of those active compounds for storage. At least in angiosperms (both the tested herbaceous and woody plants), monolignol aglycones are directly exported across plasma membranes into the cell wall. Whether there is disparate mechanism for sequestering and transporting lignin precursors in gymnosperms remains to be further clarified. (2) Vacuolar sequestration and plasma membrane transport of lignin precursors require membrane protein-mediated active transport mechanisms. Although, the possibility of a small portion of passive diffusion can’t be excluded and more than one mechanism may be involved in the transport of monolignols and their glucosides, ABC-like transporters are evidenced as the major player. In future, the particular ABC-like transporter proteins need to be identified. 

Understanding the basis for the sequestration and transport of lignin monomers and identifying the molecular components involved in those processes are significantly meaningful for manipulating plant lignification, therefore improving the digestibility of cell wall biomass. Three potential applications/consequences of the studies can be envisioned. 

(1) Elucidating the vacuolar sequestration mechanism of lignin precursors and isolating the related vacuolar transporters will enable a potential genetic engineering strategy to selectively divert lignin precursors into the “chemical sink” within the cells and reduce the amount of lignin precursors deposited into cell wall for polymerization, by which to reduce cell wall recalcitrance, meanwhile, maintain or promote the organic carbon storage in particular cell compartment (e.g., vacuole). 

(2) Similarly, exploring the mechanisms for monolignol export across plasma membrane and subsequently characterizing the potential biochemical factors involved may lead to a rational solution for controlling the deposition of lignin precursors, thus precisely manipulating both lignin content and composition in the targeted cell wall. This will enable us to alter the ratio of the labile to condensed lignin to produce more easily cleavable biopolymers. 

(3) Plant membrane transporters are not only involved in sequestering and transporting lignin precursors, but also contribute to many other biological processes in wood formation, e.g., hormone transportation and defense responses to environmental stresses. In the future, systematically identifying and characterizing the membrane-transporters involved in plant lignification will benefit both the trials to tailor lignin biosynthesis and the sustainable production of cell wall biomass. 

## Figures and Tables

The symplastic monolignols may directly export to the cell wall through passive diffusion or by active transport. Alternatively, monolignols may be sequestrated as glucoconjugates into vacuole in gymnosperms. The stored glucosides may then be transported to the cell wall and hydrolyzed to the free monolignols for polymerization.
